# Peliosis hepatis associated with hereditary haemorrhagic telangiectasia

**DOI:** 10.1093/gastro/got021

**Published:** 2013-07-23

**Authors:** F. Alessandrino, P.F. Felisaz, A. La Fianza

**Affiliations:** Foundation IRCCS, Policlinico San Matteo, Institute of Radiology, University of Pavia, Pavia, Italy

**Keywords:** peliosis hepatis, hereditary haemorrhagic telangiectasia, magnetic resonance imaging

## Abstract

Hereditary haemorrhagic telangiectasia (HHT) is an autosomal, predominantly inherited disease characterized by diffuse telangiectases involving the skin, mucous membranes, lung, brain, gastrointestinal tract and liver. Peliosis hepatis is a rare, benign disorder causing sinusoidal dilatation and the presence of multiple blood-filled lacunar spaces within the liver.

We report a case of an HHT patient with incidental magnetic resonance findings of focal hepatic peliosis.

## INTRODUCTION

Hereditary haemorrhagic telangiectasia (HHT) is a rare autosomal dominant vascular disorder characterized by angiodysplastic lesions, in which there is direct communication between arteries and veins without an intervening capillary network [1, 2].

Peliosis hepatis (PH) is a rare benign disorder causing sinusoidal dilatation and the presence of multiple blood-filled lacunar spaces within the liver. Peliosis come from the Greek *pelios*, which means bluish or gloomy, firstly used by Wagner in 1861 to describe the appearance of lesions on the cut surface of the liver [3]. The cause of PH can be related to drugs (anabolic steroids and oral contraceptives), toxins, chronic wasting diseases, infections in AIDS, haematological malignancies, diabetes and chronic alcoholism. In 20–50% of patients, no associated condition is identified [4].

We present here a case of an HHT patient with associated PH and its magnetic resonance imaging (MRI) findings.

## CASE REPORT

**Figure 1.**
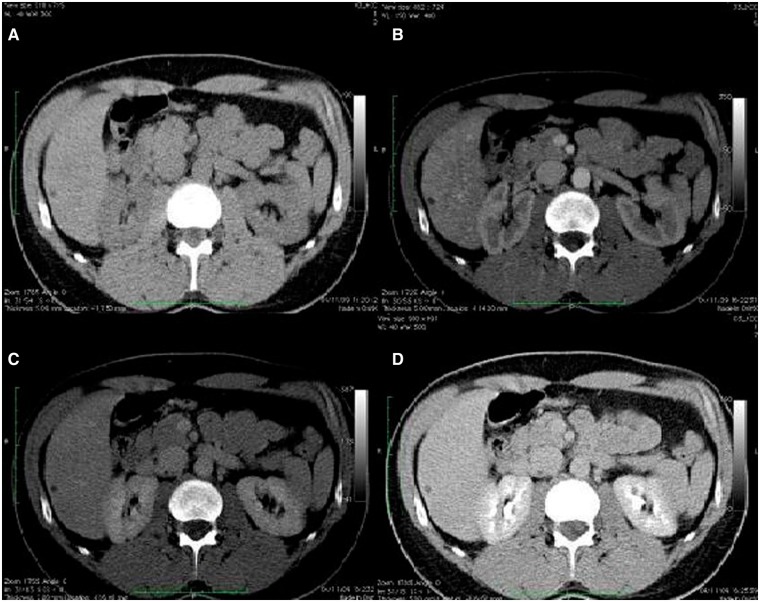
Triphasic computed tomography scan showing no focal liver lesion. Basal (A), arterial (B), portal (C) and venous phase (D) showing no focal liver lesion or no sign of diffuse liver disease except for a cyst at the left lobe of the liver.

An asymptomatic 51-year-old man came to our attention for the evaluation of two focal liver lesions incidentally detected at an ultrasound (US) examination. Three years before, the patient had already undergone a US examination and a contrast-enhanced computed tomography (CT) scan for increased liver enzymes, which showed no focal liver lesions and no sign of diffuse liver disease (Figure 1).

The patient was scheduled for an MRI. We performed a contrast-enhanced magnetic resonance imaging (CEMRI) of the liver using a Siemens MAGNETOM Symphony 1.5T (Siemens, Erlangen, Germany). Our protocol comprises a T2-weighted axial- and coronal 6 mm thick sequence with and without fat saturation, a T1-weighted gradient-echo in- and out of phase, unenhanced and dynamic gadolinium-enhanced (MultiHance, Bracco, Italy) three-Dimensional T1-weighted gradient-echo sequences.

**Figure 2.**
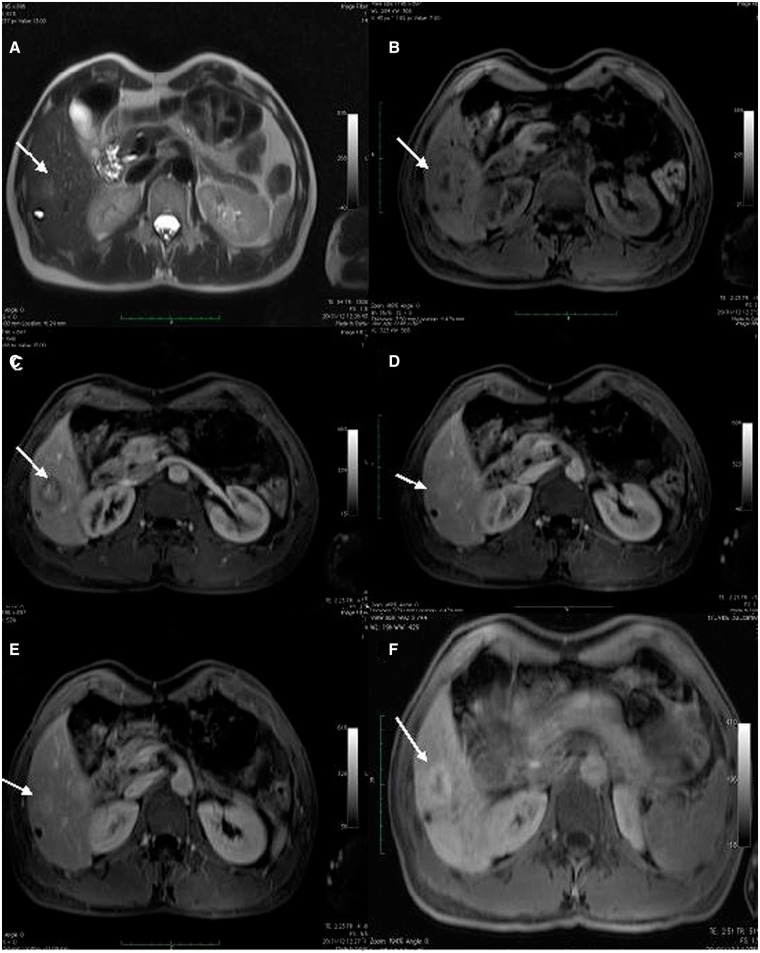
Contrast enhanced Magnetic Resonance Imaging (CEMRI), showing two focal liver lesions consistent with peliosis. T2-weighted (A), T1-weighted volume interpolated body examination (VIBE) (B), T1-weighted arterial phase (C), T1-weighted portal venous phase (D), T1-weighted late venous phase (E) and T1-weighted hepatobiliary phase (F) showing a focal liver lesion (arrows) slightly hyperintense on T2-weighted images with hypointense signal on T1-weighted images and centrifugal contrast enhancement and peripheral contrast media uptake on hepatobiliary phase.

A dose of 0.1 mmol/kg of MultiHance contrast was administered intravenously. Our examination showed three focal liver lesions in the right hepatic lobe, which appeared slightly hyperintense on T2-weighted images, centrally hypointense on T1-weighted images and showing centrifugal enhancement with a strong central contrast enhancement on delayed phases. On the hepatobiliary phase, a hypointense lesion with a hyperintense peripheral rim could be seen (Figure 2). A suspicion of focal PH was recorded.

After two months, the patient, whose mother had suffered from HHT, decided to undergo a genetic test for HHT, which turned out to be positive for mutation in HHT2-ALK1.

## DISCUSSION

HHT is an autosomal, predominantly inherited disease with variable penetrance. It is characterized by diffuse telangiectasias involving the skin, mucous membranes, lung, brain, gastrointestinal tract and liver, with a prevalence of 1–2 cases per 10 000. The prevalence of hepatic involvement in HHT has been estimated to be between 32 and 72%. Only 8% of patients are symptomatic and the severity of symptoms is related to genotype [5]. Consistent with the evidence in literature, our patient was asymptomatic and showed an HHT2 genetic type, carrying a genetic mutation involving the locus HHT2 at 12q31, which has been identified as activin receptor-like kinase 1 (ALK1)[6].

The precise pathogenesis of HHT is still undetermined, but most likely involves aberrant endothelial cell responses. It is known that ALK1 is involved in angiogenesis. Over-expression of constitutively active ALK1 can promote specific endothelial cell responses, such as proliferation, causing vascular malformations [5].

Clinical diagnosis is based on the Curacao Criteria (Table 1), according to which our patient was unlikely to suffer from HHT, showing only a first degree affected relative. His mother in fact, was diagnosed with HHT at the age of 80. Concerning liver imaging, hepatic lesions in HHT are focal or diffuse telangectases, large confluent vascular masses or hepatic perfusion abnormalities [1].

**Table 1.** Curacao Criteria

Criterion	Description
1. Epistaxis	Spontaneous recurrent nosebleeds
2. Mucocutaneous telangiectasia	Multiple at characteristic sites: fingertip pulps, lips, oral cavity
3. Visceral involvement	Gastrointestinal, pulmonary, hepatic, cerebral or spinal AVM
4. Family history	A first-degree relative affected according to these criteria

The HHT diagnosis is classified as:

***Definite***: three criteria are present.

***Possible*** or ***suspected***: two criteria are present.

***Unlikely***: fewer than two criteria are present.

PH is a rare pathological entity characterized by multiple blood-filled cavities within the liver. Two different types of disease have been described: the parenchymal type and the phlebectatic type. The former consists of irregular blood-filled cavities lined with hepatocytes, occasionally displaying liver necrosis. In the latter, blood-filled regular and spherical cavities are lined by endothelium or fibrosis [7].

Several theories concerning PH pathogenesis have been discussed: some authors proposed hepatocellular necrosis as a trigger factor for a sinusoidal dilatation, while others advocate a congenital vascular malformation or an active vessel proliferation as a possible pathogenic mechanism [4]. No genetic mutations are related to the onset of PH.

Imaging ﬁndings in PH are variable: lesions are generally hypodense before administration of intravenous contrast medium at CT. On contrast-enhanced CT, during the arterial phase, peliotic lesions typically show early globular enhancement and multiple small accumulations of contrast material in the centre of the lesions (the ‘target sign’). During the portal venous phase, a centrifugal progression of enhancement is usually observed [8, 9].

Concerning CEMRI, on T2-weighted sequences, peliotic lesions are usually hyperintense to liver parenchyma with multiple foci of high signal; on T1-weighted sequences, lesions are generally hypointense [10]. After contrast material injection, peliotic lesions usually show centrifugal enhancement. After administration of gadobenate dimeglumine, in the delayed phase, strong contrast enhancement with a branching appearance can also be observed [10, 11].

The simultaneous occurrence of HHT and PH has never been addressed before in the literature. To our knowledge, this is the first described case. More studies will be necessary to decide whether this is an incidental finding or that these diseases are somehow related. To our knowledge, genetic and aetiopathological pathways seem to be different for these diseases.

**Conflict of interest:** none declared.
